# Comparing Crop Yield, Secondary Metabolite Contents, and Antifungal Activity of Extracts of *Helichrysum odoratissimum* Cultivated in Aquaponic, Hydroponic, and Field Systems

**DOI:** 10.3390/plants11202696

**Published:** 2022-10-13

**Authors:** Nomnqophiso Zantanta, Learnmore Kambizi, Ninon G. E. R. Etsassala, Felix Nchu

**Affiliations:** Department of Horticultural Sciences, Faculty of Applied Sciences, Cape Peninsula University of Technology, P.O. Box 1905, Bellville 7535, South Africa

**Keywords:** *H. odoratissimum*, *Asteraceae*, secondary metabolites, antioxidants, aquaponics, hydroponics

## Abstract

The overexploitation of wild plants for medicinal uses and conventional agricultural farming methods, which use high amounts of water, fertilizers, and pesticides, have had devastating environmental consequences. This study aimed to evaluate the prospects of using aquaponics and hydroponics as alternative approaches to soil cultivation by comparing the crop yields, secondary metabolite contents, and the antifungal activities of ethanol extracts of *Helichrysum odoratissimum* (L.) Sweet, a widely used medicinal plant species in Southern Africa. Six-week-old *H. odoratissimum* seedlings were grown in aquaponic and hydroponic systems. The growth parameters, secondary metabolite contents, and antifungal activity against *Fusarium oxysporum* were assessed. The results for crop yield (plant height, fresh and dry weights) and the tissue nutrient contents did not change substantially (*p* > 0.05) between aquaponic and hydroponic treatments. Gas chromatography–mass spectrometry (GC–MS) analysis showed that monoterpenes and sesquiterpenes were the most abundant compounds in *H. odoratissimum*; however, no statistical difference was observed among the field, hydroponic, and aquaponic plants (DF = 2; χ^2^ = 2.67; *p* > 0.05). While there was no significant difference in polyphenol contents among the three treatments, remarkably, the flavonol contents in the leaves varied significantly (DF = 2; χ^2^ = 6.23; *p* < 0.05) among the three treatments. A higher flavonol content occurred in leaves from the hydroponic system than in leaves from the aquaponic (*p* < 0.05) and field (*p* > 0.05) systems. The MIC results showed that the ethanolic extract of *H. odoratissimum* was fungistatic against *F. oxysporum*; however, this effect was more prominent in the ethanol extracts of plants grown in the aquaponic system, with a mean MIC value of 0.37 ± 0.00 mg/mL The key findings of this study are that aquaponically cultivated plants exhibited the best antifungal activity, while higher total flavonol contents occurred in the hydroponically cultivated plants. In conclusion, aquaponics and hydroponics performed better or similar to field cultivation and are viable alternative methods for cultivating *H. odoratissimum* plants.

## 1. Introduction

The world’s population has grown rapidly in the last century, and is predicted to reach 10 billion by 2050 [1,2]. This rapid population growth severely strains natural resources, including plants, water, and land. These resources are already overexploited [3,4,5]. Anthropogenic factors, including conventional agricultural practices, are significant drivers of environmental degradation [2]. Traditional (soil-based) cultivation is often associated with high inputs of heavy inorganic fertilizers, synthetic pesticides, and water [6].

Lately, there has been an appreciation of the need to adopt environmentally friendly crop cultivation methods to mitigate environmental degradation. Aquaponics and hydroponics are increasingly used to cultivate vegetables; however, using these systems to grow medicinal plants is novel. Both cultivation technologies could limit the exploitation of some endangered medicinal plants from the wild, reduce water wastage during crop cultivation, and enhance the commercialization of medicinal plants [7]. Cultivating fish and medicinal plants in aquaponics is feasible and ecologically friendly, and integrates hydroponics and aquaculture. Aquaponics is regarded as one of the most resource-efficient production systems [8], where wastewater is recycled and reused, minimizing contamination of adjacent water bodies [9]. 

In hydroponics, another type of soilless cultivation, plants are grown in a sterile nutrient solution (water culture) and inert substrate [10]. Cultivating medicinal plants under precise and controlled environmental conditions in a hydroponic system improves quality, bioactivity, and biomass output on a commercial scale [11,12]. This approach is beneficial in areas with persistent environmental stresses, such as extreme cold, heat, and drought [11]. The prospect of using these cultivation systems to optimize the synthesis of secondary metabolites in highly valued medicinal plants is enticing [13,14]. 

Secondary metabolites produced by plants play a crucial role in plants’ defense, protection, and signaling systems [15]. Notably, many of the secondary metabolites produced by plants are valuable to the pharmaceutical industry [16]. Some bioactive compounds have pharmacological or toxicological effects on humans and animals [17]. Some plant-based compounds have cosmetics and nutritional uses; they produce drugs, dyes, fragrances, and dietary supplements [18]. Plant pathogens and predatory insects have been successfully controlled using plant extracts with high bioactive secondary metabolite contents [19,20,21]. Therefore, developing cultivation protocols to optimize the secondary metabolite content, and thus, the medicinal value of plants, is certainly a worthy research venture. 

*Helichrysum odoratissimum* (L.) Sweet belongs to the Asteraceae family, and is a spreading perennial shrub with linear, oblong leaves that are greyish-white and woolly on both sides [22]. The flowers are pale golden yellow, with tiny flower heads borne in clusters at the terminals of the branches, woody at the base, erect, and up to 50 cm high, and bloom all year [23]. It is an aromatic species, and it is widely distributed throughout intertropical and Southern Africa [24]. *Helichrysum odoratissimum* is one of the most harvested and traded plants in South Africa [22]. Many studies have validated its traditional uses in traditional medicinal treatment of abdominal pains, heartburn, fever, catarrh, headache, menstrual problems, urinary tract infections, and wounds [22,24].

Numerous compounds, including chalcone, helichrysetin, 3,5-dihydroxy-6,7,8-trimethoxy flavone, 3-0-methylquercetin, and 3’,4’,3,5-tetrahydroxy-7-methoxyflavone, are found in the flowers of *H. odoratissimum* [24,25]. The crude extracts and isolated compounds from *H. odoratissimum* exhibit pharmacological effects such as antibacterial, antimycobacterial [26,27], antifungal [22,27,28,29,30], anti-inflammatory [31], and antioxidant [26] effects, as well as cytotoxicity and toxicity [32]. 

One of the most significant problems that impedes optimal crop production is the spread of pathogens in plants. *Fusarium* wilt (*Fusarium oxysporum*) is one of the most common pests; it is widespread in nature and capable of causing significant crop and economic losses [20]. Aquaponically and hydroponically cultivated *H. odoratissimum* could be a source of active plant extracts against *Fusarium oxysporum* [21]. 

This study aimed to evaluate the prospects of using aquaponics and hydroponics as alternative approaches to soil cultivation by comparing the crop yields, secondary metabolite contents, and the antifungal activities of *H. odoratissimum* extracts cultivated in aquaponic and hydroponic systems.

## 2. Results

### 2.1. Plant Height

The results show that the heights of aquaponically and hydroponically cultivated *H. odoratissimum* plants did not vary significantly (DF = 1; χ^2^ = 1.128; *p* > 0.05) (Table 1). However, it was observed that aquaponic plants had considerably longer mean shoot lengths (17.06 cm) than hydroponic plants (15.93 cm) (Table 1).

### 2.2. Fresh and Dry Weight

When the weights of plants grown in aquaponic and hydroponic systems were compared six weeks after treatment, there was a significant difference between the *H. odoratissimum* plants cultivated in aquaponic and hydroponic treatments (DF = 1; χ^2^ = 4.84; *p* = 0.02; *p* < 0.05); the fresh weight of aquaponic plants was greater than that of hydroponic plants (Table 1). Aquaponic and hydroponic treatments did not significantly differ in the dry weights of *H. odoratissimum* (DF = 1; χ^2^ = 4.41; *p* < 0.05). Nevertheless, plants produced in the aquaponic system were heavier than plants cultivated in the hydroponic system. 

### 2.3. Tissue Analysis

#### 2.3.1. Macronutrients

The macronutrient contents, in terms of carbon, potassium, and calcium, in *H. odoratissimum* did not differ significantly between aquaponic and hydroponic treatments: C (DF = 1; χ^2^ = 2.68; *p* > 0.05), K (DF = 1; χ^2^ = 2.14; *p* > 0.05), and Ca (DF = 1; χ^2^ = 2.24; *p* > 0.05) (Table 2). However, the macronutrient contents of N, P, and Mg, on the other hand, were significantly different (*p* < 0.05) among aquaponic and hydroponic plants. The highest mean tissue nutrient contents in plants were obtained in plants grown in the hydroponic system: N (16,000 mg/kg), P (3400 mg/kg), and Mg (1950 mg/kg). Broadly, plants grown in the hydroponic system had higher tissue contents of macronutrients than aquaponically cultivated plants.

#### 2.3.2. Micronutrients

Plants grown in the aquaponic system had significantly higher leaf tissue micronutrient contents for the majority of elements, Mn (DF = 1; χ^2^ = 8.54; *p* < 0.05), Cu (DF = 1; χ^2^ = 185.41; *p* < 0.05), and Zn (DF = 1; χ^2^ = 71.12; *p* < 0.05), analyzed than those in the hydroponic system, which, on the other hand, yielded significantly higher (DF = 1; *p* < 0.05) tissue contents for B and Fe (Table 3).

### 2.4. Secondary Metabolites (Polyphenols and Flavonol)

When the three cultivation methods were evaluated, there was no significant difference (DF = 2; χ^2^ = 4.25; *p* = 0.07) in total polyphenol contents (mg GAE/g) in the leaves of *H. odoratissimum* (Table 4). However, plants cultivated via hydroponics had higher polyphenol contents (592.98 ± 76.88 mg GAE/g) when compared with the aquaponic and field-collected plants. The flavonol contents in the leaves varied significantly (DF = 2; χ2 = 6.23; *p* < 0.05) among the three treatments. Hydroponic cultivation produced a significantly higher flavonol capacity compared to aquaponic (Table 4). 

### 2.5. Antioxidant Capacity (FRAP, ABTS, and DPPH)

The FRAP analysis of *H. odoratissimum* revealed no significant effect (DF = 2; χ^2^ = 2.69; *p* = 0.14) of treatment on the antioxidant capacity of the plant extracts from the three cultivation techniques (Table 5). However, plants grown via hydroponics produced a higher mean value in the FRAP bioassay (3078.55 ± 355.44 µmol AAE/g) than aquaponic (2350.46 ± 200.18 µmol AAE/g) and field-collected plants (2196.50 ± 284.01a µmol AAE/g). In the DPPH test, which is based on the ability of an antioxidant to donate a hydrogen atom to the DPPH free radical, the extracts from the hydroponically cultivated plants displayed higher antioxidant activity; nevertheless, the DPPH contents (mol TE/g) did not vary substantially among the three cultivation methods (DF = 2; χ^2^ = 0.91; *p* = 0.4). Similarly, the ABTS capacity (µmol TE/g) results indicate that the hydroponic plants produced a significantly higher value compared to aquaponic and field-collected plants (DF = 2; χ^2^ = 8.44; *p* = 0.01) (Table 5). Broadly, *H. odoratissimum* plants cultivated in hydroponics had the best antioxidant activity.

### 2.6. Analysis of Volatile Compounds

The number of volatile compounds produced by plants did not differ substantially when the treatments (hydroponic, aquaponic, and field) were compared using the chi-square test (DF = 2; χ^2^ = 2.67; *p* = 0.26) (Table 6). The total number of compounds detected was 116 for all three cultivation methods. However, when the individual compounds were compared among treatments, the concentrations of alpha-terpinene, styrene, beta-ocimene, and cyclohexanone were significantly higher (DF = 2; *p* < 0.05) in the hydroponic plants (Table 6). Some compounds were substantially higher (DF = 2; *p* < 0.05) in aquaponic plants compared to hydroponic and field-collected plants, which included alpha-phellandrene, o-ethyltoluene, tetradecane, alpha-terpineol, alpha-curcumene, and palustrol.

### 2.7. In Vitro Fungal Activity Using the Microdilution Assay

#### The Minimum Inhibitory Concentration of *H. odorattisimum*

There was a significant difference in the minimum inhibitory concentrations among the three cultivation methods when the ethanol extracts of *H. odorattisimum* (DF = 2; χ^2^ = 7.5; *p* = 0.03) were screened against *F. oxysporum* at 18 h of incubation (Table 7). Treatment one (aquaponics) yielded the best fungistatic results, with an MIC value of 0.37 mg/mL, followed by hydroponics (0.56 ± 0.18 mg/mL). Treatment three (field-collected plants) yielded the least activity among all the treatments tested, with a MIC value of 0.75 mg/mL; this is equivalent to the synthetic fungicide (Dithane) used as a positive control, with a MIC value of 0.75 mg/mL (Table 7). 

## 3. Discussion

Many previous studies have demonstrated the promising prospects of using aquaponics and hydroponics to cultivate vegetables sustainably [33,34,35]; in addition, the current results suggest that aquaponics and hydroponics can also be used to produce high-quality medicinal plants [36]. This study’s findings show that aquaponics slightly outperformed hydroponics in plant growth (plant height, fresh and dry weight), although there were no significant differences between the two cultivation methods. These results agree with a previous study, which showed that the dry weights of *Solanum lycopersicum L.* did not significantly differ between organic and aquaponic cultivation methods [37]. Earlier, Ranawade et al. (2017) [38] studied spinach yields in hydroponic, aquaponic, and traditional (soil-based cultivation) systems, and found that aquaponically grown spinach had a higher yield than hydroponically and traditionally cultivated spinach. Additionally, Schmautz et al. (2016) [39] used tomatoes to assess whether the mineral contents and nutritional quality in plants grown in aquaponically and conventionally grown tomatoes were similar; the aquaponically grown plants were equivalent or superior to conventionally grown tomatoes. Graber and Junge (2009) [33] also reported that the plant yield of an aquaponic system was similar to conventional hydroponic production systems for three crops, aubergine, tomato, and cucumber. However, in an earlier study, Roosta and Ghorbani (2011) [40] reported that hydroponics outperformed aquaponics in many of the assessed growth parameters for two species (*Mentha sativa* and *Mentha piperita*). These authors suggested the growth of plants in aquaponic systems was likely slowed down compared to hydroponic systems because of lower concentrations of critical nutrients, such as Mn and Mg, in *Mentha sativa* shoots, and N, P, Mg, and Mn in *Mentha piperita*. 

In this study, we found no clear association between the tissue nutrient contents and the plant growth parameters. The tissue nutrient contents of C, Ka, and Ca were not significantly different between hydroponic and aquaponic plant leaves; however, the tissue macronutrient contents of N, P, and Mg were significantly higher in the hydroponic plants. Previous reports suggest that despite lower concentrations of most tissue nutrients in the aquaculture water, aquaponic plant growth results were comparable to those of hydroponics, and production can be even better than in soil cultivation [33,41,42,43]. In a recent study, researchers found that plants grown in aquaponic systems grow at the same rate or even faster than conventionally or field-grown plants [44]. This result is not unexpected given that plants have optimal thresholds for each nutrient, beyond which growth may not be positively affected. Tissue macronutrient levels in *H. odoratissimum* plants in the hydroponic system were higher for N, P, and Mg. However, this was expected because hydroponic systems have readily available nutrients in the nutrient-rich medium, unlike aquaponic systems. However, concerning aquaponic performance in terms of tissue macronutrient contents in plants, it has been proven that aquaponic systems can generate comparable concentrations of K, Ca, and Mg [34]. These elements are essential elements for plant growth [41]. 

The aquaponic system outperformed the hydroponic system in many of the plant leaf tissues’ essential micronutrients (Mn, Cu, and Zn) (Table 3). Perhaps the high concentrations of these nutrients in the aquaponics plants could be due to the high concentration of these elements in fish feed, which is routinely dissolved in the water, and then absorbed by the plants. According to Palm et al. (2018) [45], at least 50% of the nutrients in an aquaponic system come from uneaten fish feed and solid and soluble fish excretions; hence, monitoring the nutrient concentrations available for plant absorption is complex but critical. Future studies should continuously monitor the concentrations of minerals in the aquaponics system to establish the efficiency of nutrient cycling. 

When compared with aquaponic and field-collected plants, hydroponic plants had a higher polyphenol content; however, there were no significant differences among the treatments. Plants in the hydroponics yielded a considerably higher total flavonol content than the aquaponic plants and field plants, but they were also not significantly different. Broadly, these results demonstrate that aquaponics and hydroponics perform equally, or even better in the case of hydroponics, than field-cultivated plants. Research has revealed that nutrient availability can significantly influence plants’ secondary metabolism and antioxidant activity [46]. While in the current study, the high levels of Cu, Zn, and Mn in aquaponics did not positively influence the secondary metabolites, the hydroponic plants had higher N, P, and Mg, and higher flavonol contents. Previously, it has been demonstrated that these macronutrients in higher levels influence secondary metabolite production. For example, Ibrahim et al. (2010) [47] reported that nitrogen levels significantly affected the production of total phenolics and flavonoids in *Labisia pumila* Benth.; total phenolics and flavonoids were reduced with increased concentration increased progressively. 

The essential oil profiles of *H. odoratissimum* have been studied extensively, revealing that these species produce a complex bouquet of vegetative and floral volatiles [48]. Several essential oil products derived from *Helichrysum sp*. are available for medical and non-medicinal uses on the market commercially. Although there was no statistical difference in the total number of volatile constituents in the three cultivation strategies, it is worth mentioning that alpha-terpineol, a potent antioxidant and antifungal agent, occurred in higher concentrations in the aquaponic plants [49,50]. Alpha-terpineol can cause leakage of the cytoplasm and serious hyphae distortions and spore disruptions in *Aspergillus ochraceus* [51,52]. They are used as a pesticide substitute in plants because of their safety and efficiency. Other important compounds that occurred in significantly higher levels in aquaponic plants than in the hydroponic and field-collected plants included alpha-phellandrene, o-ethyltoluene, tetradecane, alpha-curcumene, and palustrol. Alpha-curcumene, isolated from the fresh aerial parts of *Senecio selloi* Spreng. DC, had high antifungal activity against *Enterobacter cloacae* [53]. In previous research, monoterpenes and sesquiterpenes. Generally, monoterpene and sesquiterpene hydrocarbons dominated the essential oil of *H. odoratissimum*. Monoterpenes, sesquiterpenes, and diterpenes are some of the broad groups of compounds present in the essential oils of most plants, including the *Helichrysum* genus; these compounds are primarily responsible for the reported antifungal, antibacterial, antidiabetic, anti-inflammatory, antiulcer, anticancer, antioxidant, antinociceptive, and antispasmodic properties associated with these plants [54]. The beauty and pharmaceutical industries extensively use alpha-terpineol (-terpineol), a monoterpenoid alcohol which was also confirmed in this study [55]. Another significant sesquiterpene found in a variety of plant essential oils known as beta-caryophyllene has been linked to several significant pharmacological effects, including immune-modulating, anti-inflammatory, anticancer, cardioprotective, hepatoprotective, gastroprotective, and renal protective effects [56]. Interestingly, the same compound was confirmed in the current study. 

The antioxidant capacity of leaves from *H. odoratissimum* cultivated via hydroponics was significantly higher (*p* < 0.05; ABTS) than plants grown in the field. Although the higher antioxidant activities were produced by hydroponic plants than aquaponic plants, the differences were not significant. Flavonols are important antioxidants in reducing oxidative damage, and have potent radical scavenging abilities [57,58,59]. Many studies on *Helichrysum* species have reported the association between flavonol and antioxidant activities [60]. The relationship between flavonol and antioxidant activities is well known; for example, Braglia et al. 2021 [37] reported that both total phenolic content (7.25 versus 6.11 mg GAEq g^−1^ DW) and antioxidant capacity (28.04 versus 20.33 mol AAEq g^−1^ DW) were significantly higher (*p* < 0.001) in aquaponic basil compared to organic soil-grown crops. On the antifungal activity, the MIC findings of this research demonstrated that aquaponic plants had significantly higher inhibition of *F. oxysporum* growth after 18 h of incubation. The reason for the higher antifungal activity in aquaponic plants’ extracts is unclear, given the lower polyphenol and flavonol contents obtained in this study. However, it is worth noting that higher concentrations of volatile compounds, such as alpha-curcumene and alpha-terpineol, with proven antifungal activity, occurred in the aquaponics plants. Additionally, these findings corroborate those from a comprehensive study of tea leaves that evaluated the link between tissue nutrient contents and secondary metabolite contents and concluded that increases in N, P, or K beyond a target value resulted in decreases in secondary metabolite concentrations [61].

## 4. Materials and Methods

### 4.1. Research Design

Four-week-old rooted cuttings of *H. odoratissimum* were grown using two cultivation systems (hydroponic and aquaponic), representing two treatments. Data on plant growth, secondary metabolite contents, and antifungal activity were obtained at the end of the experiment. The secondary metabolite contents and antifungal activities of plants obtained from aquaponics and hydroponic systems were compared with field-cultivated plants. *Helichrysum odoratissimum* seedlings were acquired from Shadowlands Wholesale Nursery Pty. Ltd. in Zevenwacht Link Road, Kuilsriver 7580, Western Cape. Plant specimens were mounted and deposited in the Herbarium of the Department of Horticultural Sciences, Cape Peninsula University of Technology (CPUT), Bellville campus, Cape Town. The roots were carefully cleaned and separated to eliminate potting soil debris. The plants were laid out on a cement floor and arranged in a completely randomized design inside the research greenhouse, where they were exposed to natural sunlight entering through the polycarbonate ceiling of the greenhouse. 

### 4.2. Greenhouse Experiment

The experiment was conducted in a greenhouse on the Bellville campus of the Cape Peninsula University of Technology (CPUT). Plants were cultivated using aquaponic and hydroponic techniques. For the hydroponic system, 4-week-old rooted seedlings of *H. odoratissimum* were transplanted into 23 cm diameter pots containing a substrate mix of two parts pine bark, one part perlite, and one part vermiculite. Fifteen replicates of *H. odoratissimum* seedlings were used. The plants were watered daily using 400 mL of deionized water and supplied with Nutrifeed fertilizer (Starke Ayres Pty. Ltd., Cape Town, South Africa), consisting of the following ingredients: nitrogen (65 mg kg^−1^), phosphorus (27 mg kg^−1^ ), potassium (130 mg kg^−1^), calcium (70 mg kg^−1^), copper (20 mg kg^−1^), sulfur (75 mg kg ^−1^), boron (240 mg kg^−1^), magnesium (240 mg kg^−1^), and zinc (240 mg kg^−1^). The fertilizer was mixed with deionized water at a dosage of 10 g/5 L. Each plant received 100 mL of the nutritional solution fortnightly, with a pH of 6.5 and an EC of 2 mS cm^−^^1^, using Milwaukee EC 50 and pH 55 kits supplied by Spraytech Pty. Ltd., Cape Town, South Africa). A recirculating aquaponic system was used in the aquaponic system. The system consisted of a fish tank containing a pump and plant grow beds (four black 50 L plastic containers with perforated lids to fit the net pots). The aquaponic system had four fish tanks containing 400 L of water each and a submersible pump that pumped the wastewater (nutrient-rich water) from the tank to a grow bed through a PVC pipe. The grow bed had a deep culture design. It consisted of two black 50 L plastic containers with a perforated lid to fit the net pots. Fifteen *Helichrysum* seedlings were transplanted into net pots containing a mixture of perlite and coco coir (50:50 ratio) as substrate. The plants were watered from the bottom through the drain holes in the net pots immersed in the nutrient-rich water pumped from the fish tank. Recycled water from the grow bed returned to the fish tank. Thus, recycling of the nutrient water was continuous. An air pump (Regent 7500) connected to an air stone using tubing was used to improve dissolved oxygen at 150 L/H in the fish tank. Each grow bed had 15 seedlings fed from the same fish tank. Ten-to-fifteen-centimeter Goldfish fingerlings (*Carassius auratus*) and fish food (Koi and Goldfish powder, small pellets) supplied by Stodels Nursery Pty. Ltd., Doncaster Road, Kenilworth 7708, Western Cape, South Africa, were used in this study. Twenty Goldfish (*Carassius auratus*) were placed in each tank (1000 L capacity). The constituents of the fish meal were maize, rice, wheat, wheat germ, dehulled soybean meal, lysine, methionine, lime, dicalcium phosphate, vitamins (A, D, E, K, BI, B2, B6, B12), biotin, folic acid, inositol, minerals, colorants, spirulina, immune stimulants, vegetable fats, natural antioxidants, and betaine. The fish were fed twice daily at 08:30 am and 4:00 pm. The aquaponic setup was replicated four times. The experiment lasted for 6 weeks. At the end of the experiment, plant height (cm) and fresh and dry weights (g) of aquaponically and hydroponically produced plants were recorded. The growth parameters of field plants were not assessed because the plants were already cultivated and established on the premises of the Bellville campus of CPUT before the commencement of the study. The harvested plant materials were used for tissue nutrient and secondary metabolite content analysis, and were screened for antifungal activities. The leaves of randomly selected field *H. odoratissimum* plants were harvested, secondary metabolites were characterized, and antifungal activity was assayed. The greenhouse conditions were 15–26 °C and 74% RH. The EC level of the nutrient solution in the fish tank was 0.8 mS cm^−1^ and the pH was 6.3. The secondary metabolite contents and antifungal activity of plants obtained from the aquaponic and hydroponic systems were compared with the field-cultivated plants. 

### 4.3. Plant Tissue Analysis

Fresh aerial plant materials (leaves) obtained from the aquaponic and hydroponic systems were sent to a certified commercial laboratory (Bemlab Pty. Ltd. in Somerset West, South Africa) for the analysis of macroelements and microelements. Aerial parts (leaves) of plants were washed with Teepol solution, rinsed in deionized water, and dried in an oven at 70 °C overnight. The dried leaves were then powdered and ashed at 480 °C for extraction using filter paper in a 50:50 HCl solution [62]. The concentrations of potassium (K), phosphorus (P), calcium (Ca), magnesium (Mg), sodium (Na), manganese (Mn), iron (Fe), copper (Cu), zinc (Z), and boron (B) were measured in the extracts [62,63]. Total combustion in a Leco N analyzer was used to determine the total nitrogen contents of the leaves. A conversion factor of 10,000 was used to convert the amounts of N, P, K, Ca, and Mg from percentages to mg/kg [64]. Three replicates from each treatment were analyzed. 

### 4.4. In Vitro Fungal Screening Using Microdilution Method

The microdilution method was used to assess the extracts’ minimum inhibitory concentration (MIC), as described by Eloff (1998) [65] and Nchu et al. (2010) [66]. Five grams of milled *H. odoratissimum* leaf materials from three replicates were extracted with 25 mL ethanol overnight, then filtered, and the filtrate was evaporated under a fan. To produce a starting concentration of 6 mg/mL, the extracts were diluted in ethanol and transferred to the first row of a 96-well microplate with wells containing 100 µL of sterile distilled water. Thereafter, the extracts were serially diluted twofold. A *Fusarium oxysporum* strain (UPFC no. 21) maintained at CPUT’s Department of Horticultural Sciences was used in the microdilution assay. Fungal conidia obtained from stock agar plates were transferred to Nutrient Broth (Merck Pty. Ltd., Cape Town, South Africa) and incubated at 25 °C for 4 h. One hundred microliters of conidial suspension (10^5^ conidia/mL) was added to each of the 96 wells of the microplates containing the plant extract. Dithane (Stodels Nursery Pty. Ltd., Garden Centre, South Africa) (200 mg/25 mL) was used as a positive control, and the negative control was the solvent blank (ethanol). Each microplate well was filled with 40 µL of 0.2 mg/mL p-iodonitrotetrazolium chloride (INT) (Sigma Aldrich SA Pty. Ltd., Kempton Park, South Africa) diluted in sterile distilled water, sealed in a plastic bag, and incubated at 37 °C and 100% RH. In the presence of fungus development, the colorless tetrazolium salt was reduced to a red-colored formazan product. 

At 18 h of incubation of the microtiter plates, the MIC values were recorded by visually comparing the pink color of the wells. The antifungal bioassay (MIC) included three replicates of each treatment.

### 4.5. Determination of Antioxidant Activities (FRAP, ABTS and DPPH)

#### 4.5.1. Ferric Reducing Antioxidant Powder (FRAP)

The FRAP assay was carried out according to Benzie and Strain’s procedure (1996) [67]. In a 96-well microplate, 10 µL of the crude extract was combined with 300 µL FRAP reagent (0.3 µM acetate buffer, pH 3.6) (Saarchem, South Africa), 10 mM 2,4,6-tripyridyl-s-triazine (TPTZ) in 0.1 µM HCl (Sigma Aldrich SA Pty. Ltd., Kempton Park, South Africa), 20 mM iron (III) chloride hexahydrate (FeCl, 593 nm). As a standard, L-ascorbic acid (Sigma Aldrich SA Pty. Ltd., Kempton Park, South Africa) was employed at concentrations ranging from 0 to 1000 µM. The absorbance was determined. The results were represented in milligrams of ascorbic acid equivalent per gram of dry weight (milligrams of AAE/g DW). Three replicates from each treatment were analyzed.

#### 4.5.2. Antioxidant Capacity of DPPH Radicals

The DPPH free radical scavenging activities of the samples were determined according to Katalinić et al. (2004) [68]. A solution of 0.135 mM DPPH produced in a dark container was used to create the DPPH radical [69]. Approximately 300 µL of DPPH solution was combined with 25 µL of the crude extract and graded concentrations (0 and 500 µM) of Trolox standard (6-hydroxy-2,5,7,8-tetramethylchroman-2-20 carboxylic acid). After a 30 min incubation period, the absorbance at 517 nM was determined as µM/Trolox equivalent per gram of dry weight (µM TE/g DW).

#### 4.5.3. ABTS Antioxidant Capacity

The ABTS assay was carried out using the method described by Re et al. (1999) [70]. Stock solutions of 7 mM ABTS and a 140 mM potassium peroxodisulfate (K_2_S_2_O_8_) (Merck, South Africa) were used. The working solution was then produced by mixing 88 µL of K_2_S_2_O_8_ with 5 µL of ABTS solution. The two solutions were thoroughly mixed and left to react at room temperature in the dark for 24 h. The standard was Trolox (6-hydroxy-2,5,7,8-tetramethylchroman-2-20 carboxylic acid) at concentrations ranging from 0 to 500 µM. The crude extracts (25 µL) were allowed to react with 300 µL of ABTS at room temperature for 30 min before being read in a plate reader (Multiskan Thermo Scientific, version 1.00.40, Vantaa, Finland) at 734 nm at 25 °C. The results were represented as µM/Trolox equivalent per gram of dry weight (µM TE/g DW).

### 4.6. Secondary Metabolite Contents

#### Determination of Total Polyphenol and Flavonol Contents

The total polyphenol contents of dried *H. odoratissimum* samples (leaves) were determined using the Folin–Ciocalteu procedure [71]. Twenty-five microliters of aqueous extracts were mixed with 125 µL of Folin–Ciocalteu reagent (Merck Pty. Ltd., Cape Town, South Africa) in a 96-well microplate and diluted 1:10 with distilled water in a 96-well microplate. The well was filled with 100 µL of aqueous Na_2_CO_3_ (7.5%) after 5 min (Sigma Aldrich SA Pty. Ltd., Kempton Park, South Africa). The plates were incubated for 2 h at room temperature before being examined at 765 nm with a Multiskan plate reader (Thermo Electron Corporation, Waltham, Massachusetts, USA). The results are represented as mg gallic acid equivalents per gram of dry weight (mg GAE/g DW) using 0, 20, 50, 100, 250, and 500 mg/L gallic acid in 10% ethanol [71,72].

The total flavonol content of dried leaves of *H. odoratissimum* plants was evaluated using a standard of quercetin 0, 5, 10, 20, 40, and 80 mg/L in 95% ethanol (Sigma Aldrich SA Pty. Ltd., Kempton Park, South Africa). A volume of 12.5 µL of crude aqueous extracts was combined with 12.5 µL of 0.1% HCl (Merck Pty. Ltd., Cape Town, South Africa) in 95% ethanol and 225 µL of 2% HCl in the sample wells, which were incubated at room temperature for 30 min. At a temperature of 25 °C, the absorbance was measured at 360 nm. The results are represented in milligrams of quercetin equivalent per gram of dry weight (mg QE/g DW) [72]. Three replicates from each treatment were analyzed.

### 4.7. GC/MS Analysis (Headspace) and Secondary Metabolite Analysis

#### 4.7.1. Sample Preparation

Fresh plant materials (leaves) were freeze dried overnight at –80 °C. After that, 1 g was weighed into a solid-phase microextraction (SPME) vial, along with 2 mL of 12% ethanol solution at pH 3.5 and 3 mL of 20% NaCl solution. A divinylbenzene/carboxen/polydimethylsiloxane (DVB/CAR/PDMS) SPME fiber was used to analyze the headspace of all the samples (grey). Three replicates from each treatment were analyzed.

#### 4.7.2. Chromatographic Separation

To determine the relative abundance of secondary metabolites, a method reported by Matrose et al. (2021) [73] was used in the separation of volatile compounds using gas chromatography (6890N, Agilent Technologies Network) coupled to an Agilent Technologies Inert XL/CI Mass Selective Detector Analytics PAL autosampler. The separation of volatiles present in the samples was achieved using a polar ZB-WAX (30 m, 0.25 mm ID) at a flow rate of 1 mL/min, and helium was used as the carrier gas. With a 5:1 ratio, the injector temperature was kept at 250 °C. The temperature of the oven was programmed as follows: 35 °C for 6 min, then 3 °C/min to 70 °C for 5 min, then 4 °C/min to 120 °C for 1 min, and lastly, 20 °C/min to 240 °C, and maintained for 2.89 min. The Mass Selective Detector (MSD) was in full scan mode when the incident occurred. Volatile compounds exhibiting a match quality of at least 90% with the mass spectral library were identified and reported.

### 4.8. Statistical Analysis

The experimental data for the plant growth parameters (plant height, fresh and dry weight), tissue nutrient content, and secondary metabolite contents were analyzed using the Kruskal–Wallis test at *p* < 0.05 level of significance. Furthermore, multiple comparisons of the means were carried out using the Mann–Whitney test. PAST was used to carry out these computations [74], and the number of volatiles in the aquaponics, hydroponics and field plants were compared using Pearson’s chi-square test. 

## 5. Conclusions

The key findings of this study reveal that *H. odoratissimum* plants cultivated via aquaponics exhibited the best antifungal activity, while hydroponically cultivated plants yielded the highest total flavonol content and antioxidant activities of the plant extracts. The results also showed that the tissue nutrient contents varied with cultivation method. Lastly, based on the chemicals identified from GC–MS analysis, aquaponic, hydroponic, and field plants yielded the same number of compounds. Based on these findings, aquaponics and hydroponics are viable alternative methods for cultivating medicinal plants. Future studies should include the economic viability of cultivating medicinal plants using these two methods. 

## Figures and Tables

**Table 1:** Growth parameters (mean ± SE) of *H. odoratissimum* grown in aquaponic and hydroponic systems for six weeks under greenhouse conditions.

Treatments	Plant Height (cm)	Fresh Weight (g)	Dry Weight (g)
T1	17.06 ± 0.48a	9.83 ± 1.85a	4.98 ± 1.06a
T2	15.93 ± 0.45a	5.57 ± 0.53a	2.53 ± 0.28b

Values shown are mean ± S.E. Means followed by the same lowercase letters in the same column are not significantly different (*p* < 0.05) following comparison using the Mann–Whitney test for aquaponic (T1) and hydroponic systems (T2).

**Table 2:** Tissue nutrient contents of shoots (mean ± SE) of *H. odoratissimum* grown in aquaponic and hydroponic systems for six weeks under greenhouse conditions.

Treatments	Quantity (mg/kg)					
	C	N	P	K	Ca	Mg
T1	462,300 ± 2655.81a	11,350 ± 317.54a	1650 ± 28.87a	6600 ± 115.47a	6850 ± 433.01a	747.5 ± 28.33a
T2	454,350 ± 4070.32a	16,000 ± 1096.96b	3400 ± 346.41b	22,850 ± 11,113.10a	8000 ± 635.08a	1950 ± 86.60b

Means followed by the same lowercase letters in the same column are not significantly different (*p* > 0.05) following the Mann–Whitney test comparison. T1, aquaponic; T2, hydroponic.

**Table 3:** Tissue nutrient contents of shoots (mean ± SE) of *H. odoratissimum* grown in aquaponic and hydroponic systems for six weeks under greenhouse conditions.

Treatments	Quantity (mg/kg)					
	Na	Mn	Fe	Cu	Zn	B
T1	14,445± 707.25a	103.8 ± 8.78a	147.67± 25.85a	17.1 ± 0.75a	116 ± 6.93a	15.43 ± 0.60a
T2	4570 ± 300.22b	78.15 ± 0.09b	168.5 ± 22.81a	5.1 ± 0.46b	44.4 ± 4.91b	48.07± 2.63b

Means followed by the same lowercase letters in the same column are not significantly different (*p* > 0.05) following the Mann–Whitney test comparison. T1, aquaponic; T2, hydroponic.

**Table 4:** Mean ± SE total polyphenol (mg GAE/g) and flavonol (mg GAE/g) contents of leaves of *H. odoratissimum* grown using different cultivation methods.

Treatments	Polyphenols (mg GAE/g)	Flavonols (mg QE/g)
T1	434.46 ± 27.67a	102.42 ± 10.27a
T2	592.98 ± 76.88a	172.8 ± 19.07b
T3	358.15 ± 58.75a	147.9 1 ± 12.01ab

Means followed by the same lowercase letters in the same column are not significantly different (*p* > 0.05) following the Mann–Whitney test comparison. T1, aquaponic; T2, hydroponic; T3, field.

**Table 5:** Mean ± SE of FRAP (µmol AAE/g), ABTS (µmol TE/g), and DPPH (µmol TE/g) contents of leaves of *H. odoratissimum* grown using different cultivation methods.

Treatments	FRAP (µmol AAE/g)	ABTS (µmol TE/g)	DPPH (µmol TE/g)
T1	2350.46 ± 200.18a	3163.67± 209.76ab	1639.13 ± 50.86a
T2	3078.55 ± 355.44a	4163.4 ± 344.29b	1907.1 ± 230.34a
T3	2196.50 ± 284.01a	2836.2 ± 86.15a	1902.7± 146.29a

Means followed by the same lowercase letters in the same column are not significantly different (*p* > 0.05) following comparison using the Mann–Whitney test. T1, aquaponic; T, hydroponic; T3, field.

**Table 6:** Volatile compounds in *H. odoratissimum* plants grown in aquaponic, hydroponic, and field systems.

* Compounds	Aquaponics Peak Area in the Chromatogram	Hydroponics Peak Area in the Chromatogram	Field Plants Peak Area in the Chromatogram	Retention Times
Decane	0.88 ± 0.02a	1.28 ± 0.33a	0.92 ± 0.01a	5.48
Alpha-pinene	3.31 ± 0.00a	24.33 ± 6.09b	20.06 ± 0.78ab	5.79
Nonadecane	0.21 ± 0.10a	0.61 ± 0.14a	0.42 ± 0.07a	6.68
Camphene	0.21 ± 0.12a	1.15 ± 0.48b	0.37 ± 0.01a	7.01
4-Methyl-octane	0.60 ± 0.30a	2.69 ± 0.94b	1.03± 0.11a	7.38
Beta-pinene	0.79 ±0.06a	2.68 ± 0.93b	1.03 ± 0.11a	8.13
Undecane	0.53 ± 0.05a	0.43 ± 0.00a	0.42 ± 0.00a	8.34
Alpha-phellandrene	2.26 ± 1.11b	0.31 ± 0.15a	0.48 ± 0.04a	8.47
Myrcene	0.77 ± 0.38a	3.22 ± 1.03b	1.38 ± 0.09a	9.59
Beta-phellandrene	0.33 ± 0.03a	0.50 ± 0.04a	0.51 ± 0.01a	12.28
1,8-Cineole	4.33 ± 0.04a	6.90 ± 0.36b	5.83 ± 1.71a	12.9
O-ethyltoluene	2.13 ± 1.17b	0.31 ± 0.03a	0.18 ± 0.10a	12.19
Ocimene	1.40 ± 0.50a	6.54 ± 2.15b	2.53± 0.28a	14.17
Gamma-terpinene	1.42 ± 0.57a	6.443 ± 2.05b	2.79 ± 0.37a	11.87
Styrene	0.22 ± 0.10a	0.67 ± 0.13b	0.45 ± 0.02a	14.72
Beta-ocimene	0.24 ± 0.09a	0.66 ± 0.13a	0.44 ± 0.03a	14.72
Para-cymene	5.18 ± 1.86a	14.76 ± 4.69b	9.16 ± 0.87ab	15.24
1,2,3-Trimethylbenzene	0.01 ± 0.01a	5.91 ± 0.92b	–	9.13
Alpha-fenchene	0.22± 0.12a	1.38 ± 0.58a	0.32 ±0.10a	15.66
Alpha-terpinolene	0.89 ± 0.20a	2.44 ± 0.46b	1.29 ± 0.13ab	15.76
Cyclohexanone	2.37 ± 0.32b	1.07 ± 0.08a	4.00 ± 0.06c	15.76
2,6,6-Trimethylcyclohexanone	0.01 ± 0.01a	0.10 ± 0.00a	0.16 ± 0.01a	15.99
3-Hexenyl acetate	1.72 ± 0.86a	3.07 ± 0.56b	1.47 ± 0.43a	14.15
2-Heptenal	1.56 ± 0.56a	3.19 ± 0.45b	1.52 ± 0.41a	11.6
6-Methyl-5-hepten-2-one	1.07 ± 0.21a	1.74 ± 0.23bc	2.31 ± 0.13c	17.41
Allo-ocimene	2.77 ± 0.05a	3.33 ± 0.47a	3.19 ± 1.84a	19.19
Octenyl acetate	2.32 ± 1.09a	1.67 ± 1.67a	4.98 ± 0.62b	16.27
3-Hexenol	0.21 ± 0.11a	0.49 ± 0.05a	0.62 ± 0.15a	19.74
4-Methyl-1,5-heptadiene	0.28 ± 0.05a	0.04 ± 0.01a	0.09 ± 0.01a	20
3-Octanol-IStd	–	–	–	
3-Ethyl-o-xylene	0.48 ± 0.16a	0.62 ± 0.24a	0.20 ± 0.00a	20.83
Para-cymene	0.90 ± 0.09a	0.96 ± 0.06a	0.96 ± 0.10a	20.96
Tetradecane	2.67 ± 0.46a	1.49 ± 0.85a	1.03 ± 0.12a	21.05
1-Octen-3-ol	4.07 ± 0.78a	14.15 ± 1.71c	7.59 ± 1.01b	21.74
Beta-fenchyl acetate	3.79 ± 0.79a	13.77 ± 1.69b	3.77 ± 0.03a	21.9
6-Methyl-5-hepten-2-ol	0.30 ± 0.00a	2.67 ± 0.38b	4.59 ± 0.05c	22.12
2,5-Dimethyl-p-xylene	0.51 ± 0.03a	2.17 ± 1.16ab	4.67 ± 0.07b	22.28
Alpha-ylangene	6.25 ± 0.01a	13.29 ± 3.98b	8.15 ± 0.63ab	22.63
Italicene	2.93 ± 0.65a	4.64 ± 1.08b	23.43 ± 0.85c	22.99
Benzaldehyde	0.23 ± 0.09a	1.15 ± 0.26b	26.18 ± 0.78c	23.16
Allyl isopentanoate	10.08 ± 1.34ab	12.70 ± 1.05b	5.24 ± 0.39a	23.66
Gamma-curcumene	4.83 ± 0.20b	2.83 ± 1.23a	22.09 ± 2.19c	20.05
L-linalool	6.71 ± 0.68a	5.18 ± 2.09a	52.53 ± 6.80b	24.32
Alpha-copaene	7.54 ± 0.91a	13.07 ± 3.84b	55.57 ± 8.76c	24.37
Sabinene hydrate	0.01 ± 0.01a	0.01 ± 0.00a	1.08 ± 0.04b	24.63
E,E-alpha-farnesene	2.29 ± 0.17a	4.67 ± 0.87b	3.45 ± 0.06ab	24.96
Fenchol	1.40 ± 0.14a	2.03 ± 0.60a	2.28 ± 0.01a	25.13
Beta-caryophyllene	115.48 ± 24.27b	114.71 ± 37.55ab	102.57 ± 20.13a	25.71
(+)-Aromadendrene	4.00 ± 0.46a	49.89 ± 25.87ab	97.52 ± 12.59b	25.8
Delta-elemene	0.66 ± 0.23a	2.37 ± 0.61b	2.06 ± 0.02ab	25.84
(-)-Isoledene	1.50 ± 0.31a	2.35 ± 0.44a	2.15 ± 0.19a	26.59
Ethyl-caprate	1.05 ± 0.10a	2.23 ± 0.45a	1.52 ± 0.11a	26.87
Pinocarveol	0.63 ± 0.01a	2.15 ± 0.34b	0.59 ± 0.03a	27.04
Gamma-elemene	0.09 ± 0.03a	0.14 ± 0.07a	0.38 ± 0.04b	27.11
Alpha-humulene	63.10 ± 1.97a	110.20 ± 28.20b	83.32 ± 7.45ab	27.74
Linaly-propanoate	27.80 ± 15.17b	100.91 ± 23.74c	7.35 ± 0.41a	27.81
Alpha-humulene	57.50 ± 3.19a	99.98 ± 24.27c	74.61 ± 9.23b	20.23
Acoradiene	28.411 ± 15.58b	101.85 ± 23.19c	6.4 8± 0.37a	27.91
1,8-Menthadien-4-ol	4.11 ± 0.05a	5.25 ± 0.66a	12.61 ± 2.79b	28.09
Beta-himachalene	–	0.73 ± 0.21a	0.44± 0.04a	28.14
Beta-himachalene	71.11 ± 35.55a	78.89 ± 39.44b	–	–
Alpha-terpineol	140.01 ± 19.25c	114.10 ± 2.44b	103.51 ± 2.56a	19.05
Ledene	250.73 ± 112.38c	232.02 ± 65.63b	343.67 ± 40.25a	28.65
(+)-2-Carene	369.88 ± 2.36a	538.97 ± 44.64c	508.44 ± 21.85b	28.73
Valencene	181.80 ± 104.35c	302.16 ± 173.95b	0.88± 0.08a	24.09
Alpha-gurjunene	1.02 ± 0.39a	1.19 ± 0.25a	0.36 ± 0.03a	29.08
Eremophilene	1.02 ± 0.39a	1.19 ± 0.25a	0.36 ± 0.04a	29.11
Beta-selinene	28.68 ± 2.82a	44.48 ± 10.06c	32.62 ± 3.45b	29.11
Neryl acetate	27.44 ± 2.10a	45.58 ± 10.74c	32.62 ± 3.45b	29.93
Alpha-bisabonele	8.84 ± 1.15a	16.43 ± 3.69b	8.63 ± 0.95a	29.31
Beta-bisabonele	6.76 ± 0.57a	12.93 ± 2.74c	8.58 ± 1.22b	29.37
Alpha-cedrene	5.89± 1.10a	15.72 ± 4.63c	10.33 ± 1.58b	29.54
7-Epi-alpha-selinene	17.24 ± 2.03a	25.47 ± 4.27b	22.90± 2.07ab	29.93
Delta-cadinene	17.24 ± 2.03a	25.37 ± 4.33b	22.90 ± 2.07ab	30.24
Alpha-curcumene	176.39 ± 24.57b	100.28 ± 41.98a	112.44 ± 50.42ab	30.24
Ar-curcumene	180.67 ± 26.09a	213.66 ± 20.00c	192.62 ± 5.33b	30.57
Gamma-selinene	2.59 ± 0.63a	62.51 ± 33.73c	6.42 ± 0.15b	30.62
Alpha-cadinene	1.68 ± 0.21a	2.63 ± 0.67a	1.92 ± 0.30a	30.95
Nerol	0.33 ± 0.03a	0.91 ± 0.16a	0.58 ± 0.06a	31.06
2-Phenylethyl acetate	4.54 ± 0.01ab	5.93 ± 1.30b	2.32 ± 0.40a	31.18
Isogeraniol	0.42 ± 0.03a	2.26 ± 0.41b	0.83 ± 0.04a	31.29
Beta-damascenone	0.42 ± 0.03a	2.26 ± 0.41b	0.79 ± 0.06a	31.29
1S-calamenene	5.48 ± 0.34c	0.34 ± 0.33a	4.13 ± 1.59b	31.5
Carveol	5.64 ± 0.38a	8.72 ± 1.68b	6.16 ± 0.36ab	31.61
P-cymen-8-ol	1.93 ± 0.66a	0.92 ± 0.15a	8.06 ± 0.55b	31.85
4-Phenyl-2-butanone	0.83 ± 0.04a	0.75 ± 0.09a	0.61 ± 0.07a	31.87
Ethyl laurate	2.04 ± 1.14a	5.39 ± 1.75b	2.97 ± 1.36a	31.98
(E)-Geranyl acetone	2.91 ± 0.73a	5.52 ± 1.83b	5.00 ± 0.35ab	32.02
Ascaridole	2.68 ± 0.78a	5.57 ± 1.84b	4.87 ± 0.32ab	32.02
Benzyl alcohol	0.72 ± 0.07a	1.42 ± 0.26a	1.20 ± 0.03a	32.24
4-Ethyl-o-xylene	2.08 ± 0.22a	3.21 ± 0.70a	2.49 ± 0.37a	32.24
Ethyl-3-phenylpropionate	2.15 ± 0.25a	3.24 ± 0.73a	2.69 ± 0.37a	32.4
Phenylethyl alcohol	4.80 ± 0.72ab	6.57 ± 1.13b	3.19 ± 0.70a	32.4
Alpha-calacorene	12.86 ± 1.61a	21.67 ± 5.27c	17.73 ± 1.19b	32.8
Palustrol	3.38 ± 0.08a	3.20 ± 0.40ab	0.82 ± 0.05a	32.92
Alpha-cubene	2.70 ± 0.30a	3.47 ± 0.74a	2.80 ± 0.31a	33.23
Caryophyllene oxide	2.54 ± 0.01a	2.71 ± 0.52a	3.02 ± 0.20a	33.91
(+)-Ledol	2.42 ± 0.10a	2.01 ± 0.24a	2.02 ± 0.12a	34.53
Alpha-caryophyllene alcohol	0.74 ± 0.03a	0.71 ± 0.19a	0.89 ± 0.01a	34.82
Fonenol	14.12 ± 1.15a	15.36 ± 1.07a	13.65 ± 1.81a	34.83
Longifolenaldehyde	12.54 ± 0.98a	13.88 ± 0.68a	11.63 ± 1.76a	34.87
N-benzylidenecyclohexylamine	3.49 ± 0.35a	3.47 ± 0.41a	4.47 ± 0.38a	35.01
Cyclooctanone	4.74 ± 0.01a	7.54 ± 1.97b	6.95 ± 0.72ab	35.16
Caryophyll-5-en-2-beta-Ol	1.97 ± 0.13a	1.54 ± 0.21a	3.36 ± 0.07b	35.78
T-cadinol	1.57 ± 0.01a	1.54 ± 0.21a	1.52 ± 0.14a	35.78
Eugenol	12.06 ± 0.95a	12.31 ± 1.69a	17.22 ± 1.52b	35.94
(+)-Calarene	1.20 ± 0.04a	1.76 ± 0.17b	1.36 ± 0.11a	36.13
Eudesm-7(11)-en-4-ol (Juniper camphor)	1.24 ± 0.09a	1.98 ± 0.24b	1.27 ± 0.08a	36.13
Beta-cadinene	1.39 ± 0.11a	1.55 ± 0.19a	1.41 ± 0.05a	36.19
Epi-bicyclosesquiphellandrene	1.74 ± 0.44a	1.41 ± 0.14a	2.27 ± 0.18a	36.3
Carvacrolok	2.95 ± 0.14a	3.47 ± 0.55a	3.61 ± 0.21a	36.46
Alpha-eudesmol	6.33 ± 0.61ab	0.62 ± 0.09a	8.06 ± 0.60b	36.62
Beta-eudesmol	2.27± 0.15a	2.48 ± 0.25a	2.22 ± 0.18a	36.73
Decanoic acid	2.37 ± 0.09a	4.03 ± 0.95b	3.00 ± 0.53ab	37.08
(-)-Phyllocladene	7.55 ± 0.76a	9.58 ± 0.61b	6.46 ± 1.03a	38.44
2,7-Dimethyl-1,6-octadiene	5.83 ± 0.34a	11.11 ± 3.55c	8.23 ± 1.75b	38.57
Xanthorrhizol	1.95 ± 0.29a	3.34 ± 0.64b	3.27 ± 0.38ab	40.9
Total number of compounds	116	116	116	

* Volatile compounds having a match quality of at least 90% with the mass spectral library were identified and reported. Means followed by the same lowercase letters in the same column are not significantly different (*p* > 0.05) following comparison using Pearson’s chi-square test.

**Table 7:** Anti-*F. oxysporum* activity (mean MIC ± SE) of ethanol extracts of *H. odorattisimum* plants that were cultivated in aquaponic, hydroponic, and field systems.

Treatments	MIC ± SE (mg/mL) 18 h
T1	0.37 ± 0.00b
T2	0.56 ± 0.18ab
T3	0.75 ± 0.00a
Dithane (Positive control)	0.75 ± 0.00a
Negative control	>3

Values shown are mean ± S.E. Means followed by the same lowercase letters in the same column are not significantly different (*p* > 0.05) following the Mann–Whitney test comparison. T1, aquaponic; T2, hydroponic; T3, field.

## Data Availability

Not applicable.
